# Ranking 93 health interventions for low- and middle-income countries by cost-effectiveness

**DOI:** 10.1371/journal.pone.0182951

**Published:** 2017-08-10

**Authors:** Susan Horton, Hellen Gelband, Dean Jamison, Carol Levin, Rachel Nugent, David Watkins

**Affiliations:** 1 School of Public Health and Health Systems, University of Waterloo, Waterloo, Ontario, Canada; 2 Center for Disease Dynamics, Economics and Policy, Washington, DC, United States of America; 3 Department of Global Health, University of Washington, Seattle, Washington, United States of America; 4 RTI International, Seattle, Washington, United States of America; Harvard Medical School, UNITED STATES

## Abstract

**Background:**

Cost-effectiveness rankings of health interventions are useful inputs for national healthcare planning and budgeting. Previous comprehensive rankings for low- and middle- income countries were undertaken in 2005 and 2006, accompanying the development of strategies for the Millennium Development Goals. We update the rankings using studies published since 2000, as strategies are being considered for the Sustainable Development Goals.

**Methods:**

Expert systematic searches of the literature were undertaken for a broad range of health interventions. Cost-effectiveness results using Disability Adjusted Life-Years (DALYs) as the health outcome were standardized to 2012 US dollars.

**Results:**

149 individual studies of 93 interventions qualified for inclusion. Interventions for Reproductive, Maternal, Newborn and Child Health accounted for 37% of interventions, and major infectious diseases (AIDS, TB, malaria and neglected tropical diseases) for 24%, consistent with the priorities of the Millennium Development Goals. More than half of the interventions considered cost less than $200 per DALY and hence can be considered for inclusion in Universal Health Care packages even in low-income countries.

**Discussion:**

Important changes have occurred in rankings since 2006. Priorities have changed as a result of new technologies, new methods for changing behavior, and significant price changes for some vaccines and drugs. Achieving the Sustainable Development Goals will require LMICs to study a broader range of health interventions, particularly in adult health. Some interventions are no longer studied, in some cases because they have become usual care, in other cases because they are no longer relevant. Updating cost-effectiveness rankings on a regular basis is potentially a valuable exercise.

## Introduction

“League tables,” which rank the cost-effectiveness of health interventions are a useful input for prioritizing health expenditures, especially for national health budgets. They have been used as policy tools for high-income countries, including a comprehensive analysis for Australia [1], and a similar analysis for cancer across high-income countries [2]. Some low- and middle-income countries (LMICs), such as Mexico, have also used league tables in their policymaking process [3]. For LMICs as a group, two major reviews of cost-effectiveness have informed strategies to achieve the Millennium Development Goals [4,5]. Cost-effectiveness is not the only important criterion for policy choice (e.g., sustainability, equity, and affordability also matter), but it provides a useful and comprehensible reference point.

As strategies and priorities are set for the Sustainable Development Goals (SDGs) and countries consider the transition to Universal Health Coverage (UHC), it is timely to update the previous reviews for LMICs. In this paper we synthesize the results from recent analyses in six different disease areas, to provide a comprehensive, updated comparison across a broad range of conditions, to examine changes over the past 10–12 years, and to highlight research gaps.

## Methods

A database of cost and cost-effectiveness results was constructed for the first six volumes of the *Disease Control Priorities*, *3rd edition* (*DCP-3*) [6–11]. Systematic searches were conducted in six major health areas, supplemented by expert surveys and existing published systematic surveys and reviews [12–17]. The surveys covered literature from 2000 to mid-2013 published in English, since the literature prior to 2000 has been previously reviewed [5].

The searches undertaken employed keywords associated with economic outcomes, as well as names of all LMICs and regions, and the major disease conditions relevant for each major health area. We report here the results per Disability-Adjusted Life-Year (DALY) averted. In most *DCP-3* volumes, studies were also graded according to the Drummond checklist to assess quality of the economic analysis [18]. Further details of the searches and summaries of the findings for the six major health areas are available [12–17]. Summary information about each of the 93 health interventions analyzed and full references for the 149 published studies are provided in S1 Table.

All costs were converted to 2012 US dollars by adjusting prices to 2012 in the original currency of the country concerned and then converting to US dollars using the exchange rate for 2012. One group of studies that could not as readily be converted were those where outcomes were expressed in international dollars of a World Health Organisation (WHO) region [4], since consumer price indices and exchange rates with the US dollar are not publicly available for those regional aggregates. Although methods exist to make an approximate conversion, additional information is required which is not always readily available from the original study, namely the proportion of all costs (both of the intervention itself and, where relevant, also those averted by the intervention) accounted for by tradeable and by non-tradeable inputs.

We opted to use exchange rate conversions rather than purchasing power parity (PPP) ones. Studies using the WHO-CHOICE (CHOosing Interventions which are Cost-Effective) methodology [4] have often used PPP conversions, which assume that health interventions have the same mix of tradeable and non-tradeable inputs as the economy does overall. However, health interventions vary considerably, from those involving behavior change communication by community health workers (relying heavily on non-tradable inputs) to vaccine delivery or use of rapid diagnostic tests (relying heavily on tradable inputs) and no single conversion method is perfect. We opted for the exchange rate method as it is more readily understood by non-economists, and also allows comparison with the earlier *Disease Control Priorities* work [5]. Using market exchange rates can, however, be problematic if exchange rates are “sticky” and do not respond immediately to differential rates of inflation between countries.

The cost-effectiveness rankings from individual volumes were aggregated to provide two sets of “league tables,” one for adults and one for children. In a few cases where no study using DALYs was available for an important intervention (for example, human papillomavirus [HPV] vaccination), a study using Quality-Adjusted Life Years (QALYs) is used instead, and this substitution is indicated. A natural logarithmic scale is used for cost in the figures because small differences in cost per outcome are less important for the least cost-effective interventions (i.e., those with the highest cost per outcome). For some interventions, a single study provided a point estimate for cost-effectiveness, whereas for other interventions multiple studies were available and/or the individual study provided a range of estimates. In the figures, the geometric mean of the endpoints of the range is the point estimate used. This works better for a natural log scale axis, and also is more appropriate where the ranges are very different.

WHO has issued guidelines on thresholds for acceptable costs per DALY averted. They recommend that that anything costing less than the per capita GNI per DALY averted is “very cost effective” [19] and anything costing less than three times per capita GNI is “cost-effective.” Recent research suggests that health budget constraints are too tight to be able to afford everything that is even “very cost-effective” according to the WHO threshold, and that thresholds should be lower [20]. Deriving a more appropriate threshold (e.g., using the marginal health gain with the existing health budget) requires country-specific data. A recent analysis suggests that a threshold of approximately one-half of GNI per capita would be more appropriate for LMICs than the WHO-suggested thresholds, and reflects better what people in those countries are able and willing to spend from the public budget [21].

In what follows, a lower threshold of $200 per DALY is used to identify priority interventions for consideration in low-income countries (all but four countries in the World Bank database had per capita income above $400 in 2013). A higher threshold of $500 is used to identify priority interventions for consideration in lower-middle income countries (all of which had per capita GNI above $1045 in 2013). Of course, other considerations such as equity, affordability and feasibility will also be important in priority-setting for individual countries, depending on the context.

## Results

**Fig 1:**
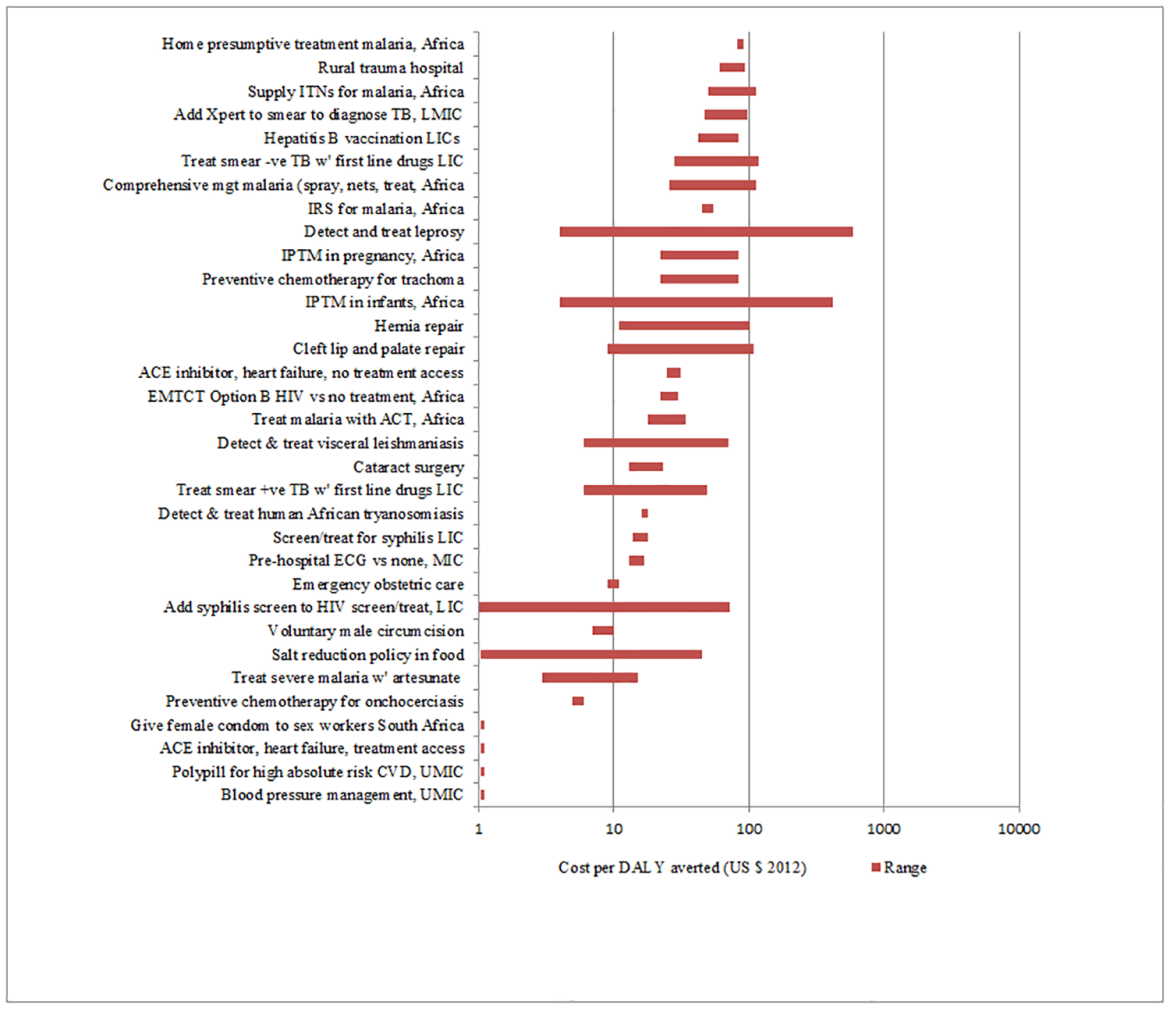
Interventions for adults costing less than $100 per DALY averted.

**Fig 2:**
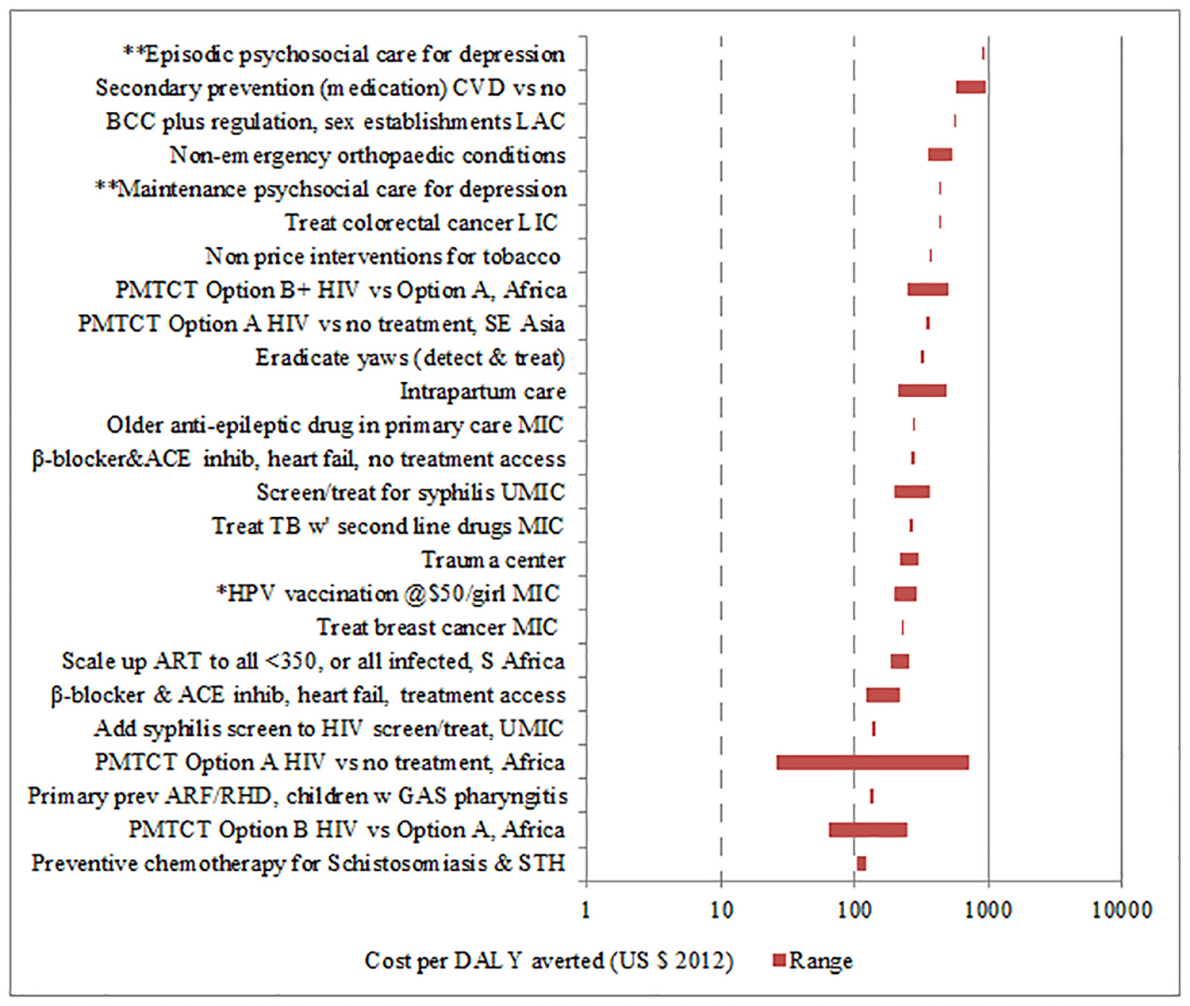
Interventions for adults costing between $100 and $999 per DALY averted. * denotes outcome in QALYs ** denotes context is primary care, UMIC.

**Fig 3:**
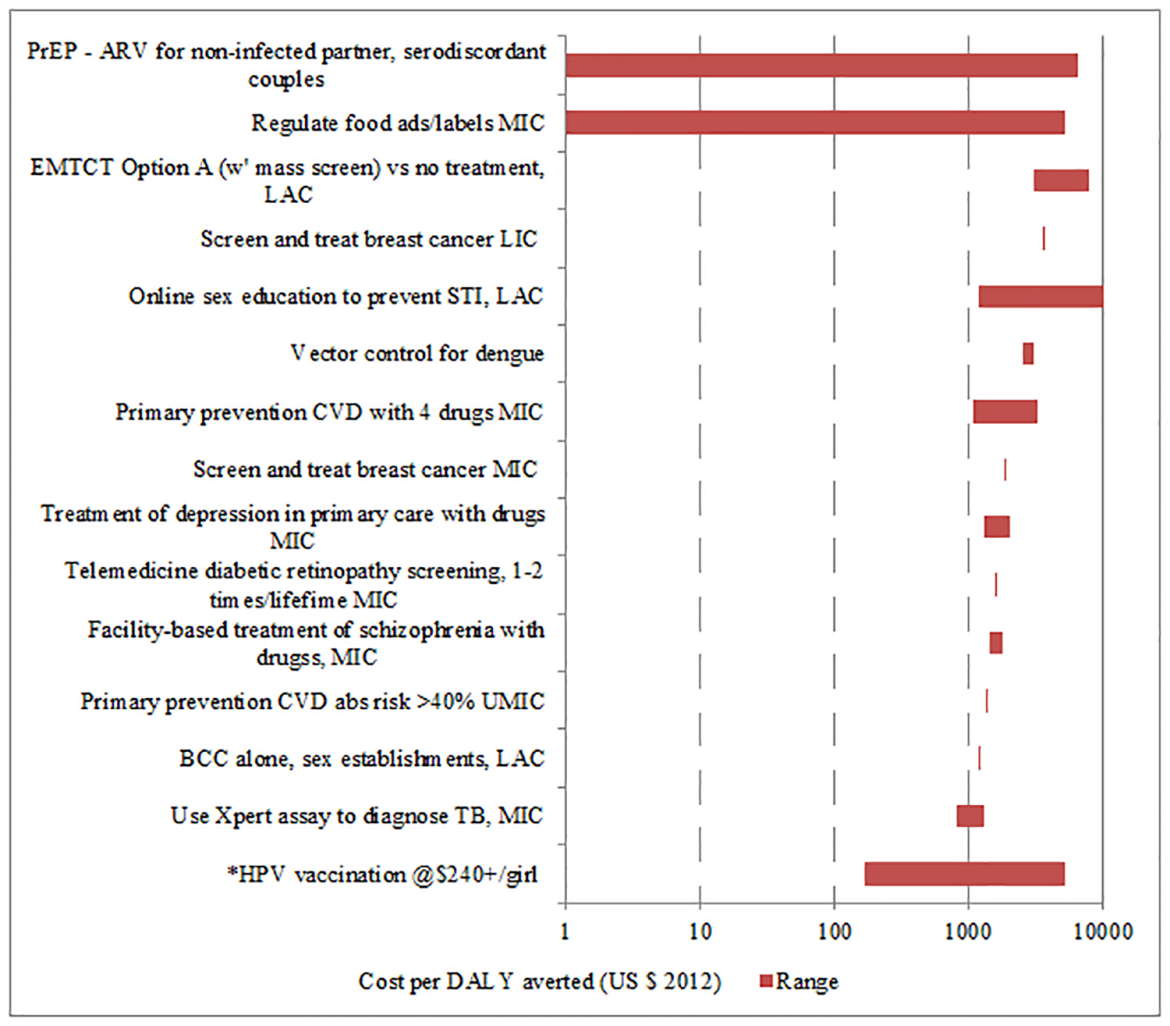
Interventions for adults costing $1000 or more per DALY averted. * denotes outcome in QALYs.

**Fig 4:**
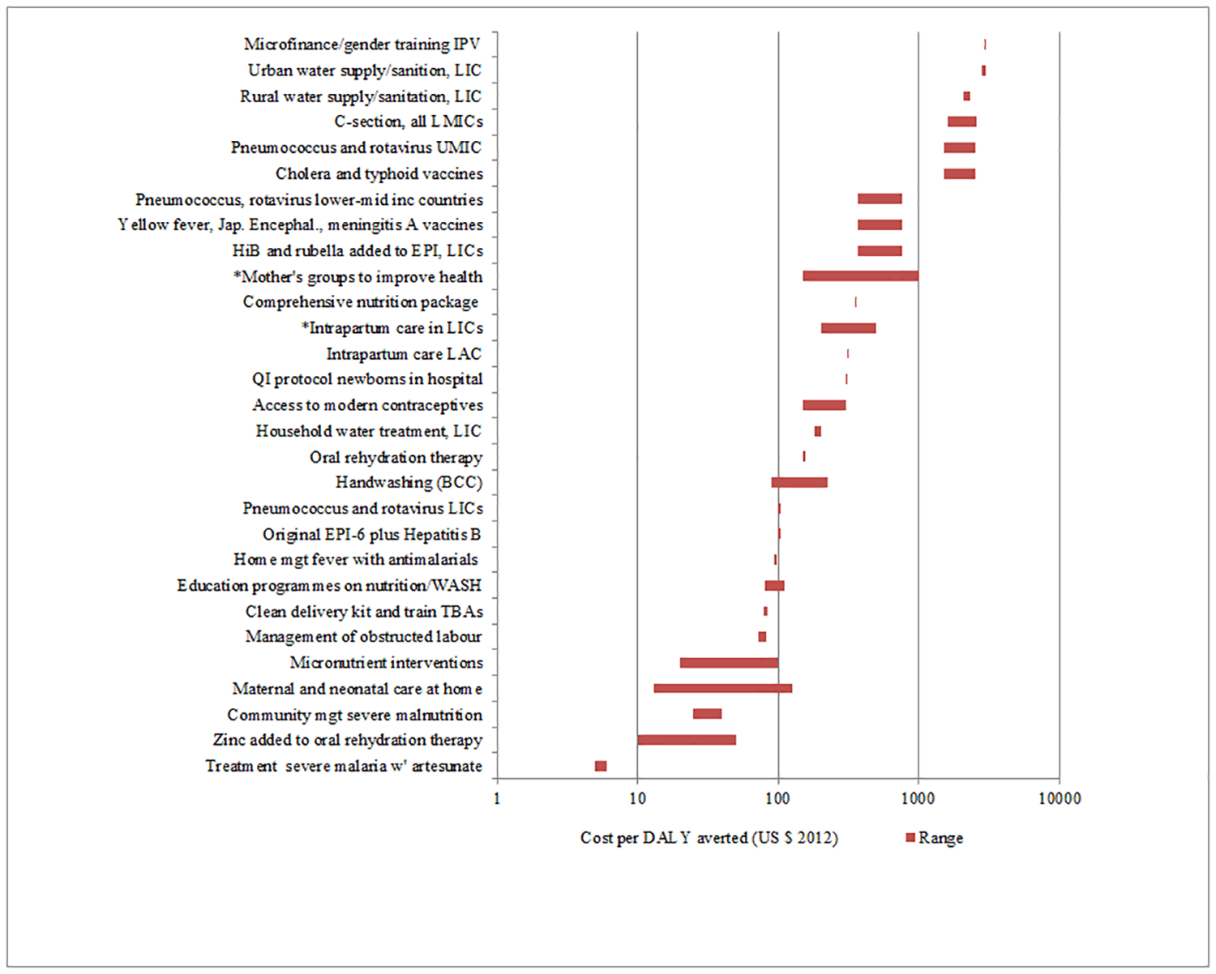
Interventions for children. * denotes outcome in QALYs.

We identified cost-effectiveness estimates for 93 interventions and contexts (Figs 1–4), drawn from 149 studies. We exclude cost-effectiveness studies of tax and subsidy policies. Although broad national policy changes are very important, it is more difficult to estimate their costs, and their cost-effectiveness is not readily compared with that of individual health interventions. Table 1 lists the notes and abbreviations used in all four figures.

**Table 1:** Abbreviations for all figures.

Abbreviation	Stands for:
ARF/RHD	acute respiratory failure/rheumatic heart disease
ARV	antiretrovirals (medication)
BCC	behavior change communication
CRC	colorectal cancer
CVD	cardiovascular disease
EMTCT	Elimination of Mother-to-Child Transmission of HIV
EPI	expanded program of immunization
HPV	human papillomavirus
IPV	intimate partner violence
GAS	group A streptococcus
GPs	general practitioners
HIB	*Haemophilus influenzae* B
IPTM	intermittent preventive treatment for malaria
IRS	indoor residual spraying (for malaria)
ITNs	insecticide-treated nets (for malaria)
IYCF	infant and young child feeding interventions (combine education with food distribution to poorest)
LIC	low income countries
Low-mid IC	lower-middle income countries
Meds	medications
MIC	middle income countries
Option A	use of single-drug regime for pregnancy for EMTCT
Option B	use of 2-drug regime for pregnancy for EMTCT
Option B+	use of 2-drug regime during pregnancy and then lifelong for women
PrEP	pre-exposure prophylaxis (for HIV)
QI	quality improvement
STI	sexually transmitted infections
TB	Tuberculosis
TBA	traditional birth attendants
UMIC	upper-middle income countries

In a few instances the same intervention appears more than once in different contexts, with different costs per DALY averted. For example the cost-effectiveness of HPV vaccination has been estimated at two different prices per vaccinated girl: the lower Gavi price (available to some lower-middle income countries) and the (usually) higher price for countries not eligible for Gavi support (e.g., upper middle-income countries). (Gavi has used its ability to undertake bulk purchases and multi-year commitments for vaccines to obtain favourable prices; however only those countries eligible for Gavi support have access to these prices, and other countries have to negotiate prices with manufacturers).

Where relevant, the economic level of the country where the study occurred is identified (for example lower-income as compared to lower-middle income country and upper-middle income country) because human resource costs differ significantly, and disease patterns are different. In other cases (particularly for HIV/AIDS), the epidemiologic context is identified. The results from southern Africa (which faces a generalized epidemic in a few countries) are different from other countries (where the epidemic is more concentrated in certain population groups). If no context is identified, the results are expected to be generally applicable in LMICs.

Of the 93 cost-effectiveness estimates, reproductive, maternal, newborn and child health contribute 37% of the interventions, and major infectious diseases (HIV-AIDS, TB, malaria, and neglected tropical diseases) 24%. This is not surprising, given that the Millennium Development Goals focused on these areas of health. Since international organizations such as the Global Fund and Gavi mobilized significant resources, there was considerable interest in (and funding for) cost-effectiveness studies in these health areas. There are far fewer economic studies for each of the other four areas considered here (surgery, cancer, mental health and cardiovascular disease).

Studies are typically done where new policy measures are being considered, e.g. new vaccines, new guidelines for treatment, new diagnostic tools. Hence, no new studies were found of well-established interventions (such as the original Expanded Program of Immunization with six vaccines). Studies of some of these established interventions exist from before 2000. In other cases, for example, emergency appendectomy, the importance of the intervention was established long before cost-effectiveness estimates became common for LMICs, so no studies are found.

More than half of the interventions in Figs 1–4 cost less than $200/DALY averted. These interventions could be considered for publicly-funded healthcare in low-income countries. These interventions include:

- Treatment of various (primarily infectious) diseases: malaria, tuberculosis (including TB that is resistant to first-line drugs), HIV-AIDS, syphilis and four of the neglected tropical diseases (NTDs); basic treatment using medication for heart failure;
- Prevention of various (primarily infectious) diseases: male circumcision, intermittent preventive treatment in pregnancy and in infants against malaria as well as insecticide-treated nets and indoor residual spraying, antiretroviral therapy for pregnant women, hepatitis B vaccinations, HPV vaccination at $50/fully vaccinated girl; pneumococcus, rotavirus and *Haemophilus influenzae* type b (Hib) vaccines in low-income countries;
- Selected basic surgical interventions: basic trauma surgery and emergency obstetric care; surgery for cataracts, hernia, cleft lip and palate; and
- Other miscellaneous interventions: training traditional birth attendants and general practitioners for births, and community-based neonatal care.

Those interventions costing $200 to less than $500/DALY averted could be considered for lower-middle income countries (in addition to the items in the list above). These include:

- Surgery for selected non-emergency orthopaedic conditions;
- Selected interventions for mental health in primary care;
- Treatment of one additional NTD; and
- Various nutrition interventions

Examples of interventions costing more than $500/DALY averted and potentially appropriate for consideration in upper-middle income countries include:

- Secondary and primary prevention of CVD with medication;
- Additional mental health interventions;
- PrEP (antiviral treatment of uninfected partners of HIV-infected individuals);
- Selected behavior-change interventions; and
- Provision of balanced protein-energy supplements in pregnancy.

## Discussion

A similar analysis to the one reported here was conducted for *Disease Control Priorities*, *2*^*nd*^ *edition* covering studies through about the year 2000 [5], providing an informative source of comparison for the current results, which date from 2000 through part of 2013. The differences are not only in the results of cost-effectiveness studies, but tellingly, in the topics studied.

About a half of the interventions appear in both the pre- and post-2000 compilations. The rest represent some significant changes. Some new interventions that were not in widespread use before 2000 have been evaluated, many of them related to substantial investments in *new technologies and new methods* to change behavior over the MDG period. For some interventions, substantial *reductions in prices* have occurred, which have made previously-unaffordable interventions less costly and hence more cost-effective. This is particularly true for vaccines where efforts by Gavi (among others) have brought down vaccine prices; and for malaria and AIDS treatments where efforts by the Global Fund and Medecins sans Frontieres (among others) have similarly brought down drug prices. Some *new areas of health*, particularly those not involving MDG targets, have been studied, making more detailed cost-effectiveness data available beyond the areas of maternal and child health and major infectious diseases. *Some interventions have changed priority*, either as the disease context has changed, or as experience has led to a revision of what was expected based on pilot programs. And finally, *some interventions no longer appear on the list*, despite being found to be cost-effective in the previous study. This may be because they have been mainstreamed and there is either no further need to estimate or update cost-effectiveness, or because they have been superseded by other more effective or more cost-effective interventions. Examples in each of these categories is given in the following sections.

### New technologies and methods

New interventions for which cost-effectiveness data have become available for LMICs include treating severe malaria with rectal or injected artesunate (which can be done prior to reaching hospital), adding the Xpert MTB/RIF assay to sputum-smear testing to diagnose disease and determine antibiotic susceptibility, and HPV vaccination for girls to prevent cervical cancer. These all fall into the <$200/DALY range, in the appropriate contexts. However other new technologies, such as PrEP, have a relatively high cost per DALY in the majority of cases.

### Changes in prices

Reduced prices of pneumococcal and rotavirus vaccines are examples of changes in costs which dramatically change the cost-effectiveness of the interventions. These were high cost per DALY averted interventions in the pre-2000 review, and at current Gavi prices for low-income countries, cost less than $100/DALY averted. Another major example is the neglected tropical diseases (NTDs). Following the 2012 London Declaration [22], the key drugs have been donated by the manufacturers, which has moved elimination of NTDs by prevention and treatment substantially up the priority list in terms of cost-effectiveness, in the past decade.

### New health areas

Efforts by the surgical community (for example the *Lancet* Commission on Global Surgery, and the first DCP-3 volume on surgery) have increased the interest in and emphasis on cost-effectiveness of surgery. Several surgical interventions both cost less than $200 per DALY averted, and can (if urgent) be implemented either in a district hospital with a general surgeon (e.g., emergency obstetric care and basic trauma care), or (if non-urgent) in a specialized facility with high volume and modest cost (cataract surgery, repair of cleft lip and cleft palate). Similar efforts are underway in the global cancer community, and one study suggests treatment of early-stage breast cancer falls in the <$200/DALY averted category for middle-income countries (although not in low-income countries, and screen-and-treat costs more than $200/DALY averted).

### Interventions that have changed priority

Adolescent health and nutrition programs in school appear as a high priority (low cost per DALY averted) in 2006, but not in 2016, because more recent studies are much more cautious about whether these programs will have long-term positive effects.

### Interventions that no longer appear on the list

Changing technology also means that some previously cost-effective interventions have been either superseded or become usual care. This is particularly evident for HIV and AIDS. In the pre-2000 compilation, eight interventions appeared in the highest-priority list: peer and education programs for high-risk groups; condom promotion and distribution; voluntary counselling and testing (without treatment); diagnosis and treatment of sexually transmitted infections; blood and needle safety, tuberculosis co-infection prevention and treatment; opportunistic infection treatment, and prevention of mother-to-child transmission were included among the most cost-effective interventions (using <$150/DALY averted in 2001 US dollars, roughly comparable to <$200/DALY averted in 2012 US dollars). A decade later, with treatment with antiretrovirals in the “highest priority” list, all but two of the other interventions fell off the list (the remaining two are prevention of mother-to-child transmission, now termed elimination of mother-to-child transmission, and testing for and treatment of other sexually-transmitted infections). Most of the interventions had become usual care, but voluntary counselling and testing without treatment had been superseded by test and treat.

A major limitation of the cost-effectiveness literature, particularly acute for LMICs, is that it is biased according to the diseases of greatest interest during the period under study. In the current study, the literature over-represents infectious conditions (and childbirth), since these have been prioritized by international donors. Drugs and vaccines tend to be over-represented relative to behavior change interventions, as manufacturers use cost-effectiveness data as part of the adoption process. Some areas of future research need are discussed in the conclusions below.

### Measurement issues

The ability to conduct a large comparative study such as this relies on use of common methodologies by individual study authors. For effectiveness studies, a lot of progress has been made applying standard guidelines for systematic reviews and by using explicit criteria for evaluating evidence. For economics studies, the fairly recent adoption of a common set of reporting standards [23], and the development of a “reference case” for conducting economic evaluations in LMICs [24], is a move in the same direction.

A bigger issue is the common metric for cost-effectiveness. The DALY has been the predominant health outcome metric used for studies of LMICs over the last decade or more. It has the advantage over the QALY for work in multiple countries in that a single set of disability weights are used across countries, whereas QALY weightings are, in theory, country specific, and generating QALY weights can be a costly process. Recent concerns about the DALY relate to the issue of discounting costs and health benefits further in the future. Although this is very much accepted by economists, some health specialists find it more problematic. The Institute for Health Metrics and Evaluation in some recent work has moved to using undiscounted DALYs to measure global burden of disease [25] but without using a new term to differentiate these undiscounted DALYs. This is guaranteed to cause confusion.

The DALY measure itself has some limitations. Using the DALY measure tends to underrepresent interventions where outcomes are not readily measured in this metric, such as family planning, and interventions in nutrition where the outcomes are improved cognition rather than improved health.

On the cost side, studies predominantly use market exchange rates to compare across different currencies. However an influential body of work from the WHO, the WHO-CHOICE study, used international dollars for WHO subregions rather than countries. International dollars make cross-country comparisons somewhat easier to understand by adjusting for salary differences as a component of costs. The downside is that international dollars make comparison more difficult with other studies not using international dollars. It is not simply a matter of using the US $/purchasing power parity (PPP) exchange rate, since it is necessary also to have information about cost structure. A further complication is that there are no published indices for PPP exchange rates of regions.

The big advantage of WHO-CHOICE was the ability to compare many interventions at one time, when the MDG strategies were being evaluated, and to compare the outcome of combinations of interventions. The disadvantage is that funding to replicate such a large comprehensive evaluation is hard to achieve. Using simpler methods (e.g., market exchange rates) allows the synthesis of many smaller individually-directed studies.

## Conclusions

Cost-effectiveness is not the only criterion on which to choose health priorities, however it is useful for identifying what is given up when a less cost-effective intervention is prioritized. It is also a useful tool for advocacy for increased health budgets. This survey has used cost-effectiveness measures from several hundred studies for LMICs to help identify candidates for priority health packages, which may assist policymakers considering how to move to universal healthcare coverage. Comparisons with a similar analysis just over a decade ago demonstrate the degree of change that has occurred.

This survey has identified some of the gaps where future research on cost-effectiveness is needed. Given the ongoing decline in infectious disease burden and the growing burden of NCDs, more analyses for NCDs are needed for LMICs. It will not be possible to achieve the aim of health convergence within a generation without initiating interventions to reduce NCDs (where the lag between intervention and outcomes is often much longer than for infectious diseases). The survey highlights the lack of any study of cost-effectiveness for childhood cancer and the dearth of information on cost-effective interventions for mental health in LMICs. Another area for future work includes the cost-effectiveness of resource-appropriate treatment of early-stage cancers (such as breast and cervical). Given the growth of obesity worldwide, cost-effectiveness studies of interventions to change patterns of diet and inactivity in urban areas are needed. A new (as of March 2016) publicly-available online global database of cost-effectiveness studies using DALY outcomes will make future updates easier [26].

The major changes in ranking of health priorities over the past decade, underscore the need for periodic repetition of “league table” exercises such as this.

## Supporting information

S1 Table Details of interventions included in Figs 1, 2, 3 and 4, ordered by increasing cost per DALY averted. (DOCX)
