# Supplementary material for: Ranking 93 health interventions for low- and middle-income countries by cost-effectiveness
Source: PLoS One. 2017 Aug 10;12(8):e0182951. doi: 10.1371/journal.pone.0182951 (PMC5552255; doi:10.1371/journal.pone.0182951)
Supplement: S1 Table — (DOCX) [file pone.0182951.s001.docx]

**Supplementary Table 1. Details of interventions included in Figures 1, 2, 3 and 4, ordered by increasing cost per DALY averted.**

| **Intervention** | **Cost-effective-ness US $ of 2012/DALY** | **Brief summary of details (unless otherwise specified, comparator is “usual care”)** |
| --- | --- | --- |
| **Figure 1** | | |
| Blood pressure management,^1^ UMIC | Cost-saving | A customized, guideline-oriented training program for primary healthcare providers was conducted in four community health centres in China, 140 hypertensive patients were recruited and followed-up for 12 months (urban, rural) |
| Polypill for high absolute risk CVD^2^, UMIC | Cost-saving | Treatment with four drugs for people with an absolute cardiovascular risk of more than 20% at 10 years in Argentina |
| ACE inhibitor vs no medication, heart failure, with access to treatment^3^ | Cost-saving | Regimen consisting of a calcium- channel blocker, and an ACE-inhibitor was given to patients older than 55 years without a history of CVD to prevent CVD, with access to treatment |
| Give female condom to sex workers^4^, South Africa | Cost-saving | Distribute female condoms among commercial sex workers and their clients in South Africa |
| Preventive chemotherapy for onchocerciasis^5,6^ | 9 | Annual invermectin treatment |
| Treat severe malaria with artesunate vs quinine^7,8^ | 5 | Use of parenteral artesunate to treat children with severe malaria in Africa and south-east Asia versus refer |
| Salt reduction policy in food ^2,9,10^ | Cost-saving to 45 | Reduce salt content in processed and home-prepared food |
| Voluntary male circumcision^11–13^ | 10 | Adult male circumcision program in sub-Saharan Africa |
| Add syphilis screen to HIV screen/treat,^14^ LIC | 9 | Rapid test with immediate results and treatment vs syndromic surveillance for pregnant women |
| Emergency obstetric care^15^ | 15 | Estimates from a small hospital in Bangladesh over 3 months; used judgement to compare to outcome with no treatment; 62% of DALYs from obstetric care |
| Pre-hospital ECG vs none,^16^ MIC | 16 | Prehospital Electrocardiogram (ECG) by general practitioners in India |
| Screen/treat syphilis,^17^ LIC | 17 | ICS syphilis screening, penicillin treatment in 43 countries in Sub-Saharan Africa |
| Detect and treat human African trypanosomiasis^18^ | 22–83 | Literature review on interventions to reduce, control or eliminate Human African Trypanosomiasis |
| Treatment smear positive TB with first-line drugs,^19^ LIC | 6–49 | Literature review: Conventional health facility-based approaches to care and increased decentralization, and involvement of communities in care provision for tuberculosis (TB) |
| Cataract surgery^20^ | 6–70 | Survey of other studies: use results from nine low and lower-middle income countries |
| Detect and treat visceral leishmaniasis^21^ | 18 | Compare chemotherapy to no intervention |
| Treat malaria with ACT,^22^ Africa | 18–34 | Modelled to estimate the incremental cost-effectiveness of using artemisinin-based combination therapies to address antimalarial resistance |
| EMTCT Option B HIV versus no treatment,^23^ Africa | 26 | Use of HAART approach for prevention of mother-to child transmission in two health centers in Malawi |
| ACE inhibitor versus no medication,^3^ heart failure, no access to treatment | 28 | Regimen consisted of a calcium-channel blocker, and an ACE-inhibitor was  given to patients older than 55 years without a history of CVD to prevent CVD |
| Cleft lip and palate repair^24–26^ | 9–108 | Corlew: study of one year’s worth of patients at Kathmandu hospital;  Magee: study of eight surgical missions to four countries in one year.  Moon: study of four years of surgical missions to Vietnam. Both studies: compared to no intervention. |
| Hernia repair^27,28^ | 11–101 | Tension-free inguinal hernia repair, four hospitals in western Ghana; expert opinion used to compare to no treatment (2010 study). Same method applied to surgical mission to small hospital in rural Ecuador (2012 study) |
| Intermittent preventive treatment malaria in infants, Africa^29,30^ | 4–422 | Analysis of data for delivery of intermittent preventive treatment of malaria in infants in sub-Saharan Africa |
| Preventive chemotherapy for trachoma^31,32^ | 22–83 | Trichiasis surgery to control trachoma in sub-Saharan Africa |
| Intermittent preventive treatment malaria in pregnancy,^33^ Africa | 4–591 | Community-based delivery of intermittent preventive treatment with sulfadoxine-pyrimethamine to pregnant women |
| Detect and treat leprosy^34^ | 50 | Interventions to prevent disability due to leprosy |
| Indoor Residual Spraying for malaria,^35^ Africa | 26–112 | Two Indoor Residual Spraying programs operating on a national scale in South Africa and Mozambique |
| Comprehensive management malaria (spray+nets+treat),^36^ Africa | 28–117 | Malaria Control measures consisted of vegetation clearance, modification of river boundaries, draining swamps, oil application to open water bodies and house screening |
| Treatment smear negative TB first-line drugs,^19^ LIC | 42–84 | Literature review |
| Hepatitis B vaccination,^37–39^ LIC | 47–97 | Hepatitis B vaccination, three doses Hepatitis B vaccine @$1.08 per dose |
| Add Xpert to smear to diagnose TB,^40^ LMIC | 50–114 | Introduction of Xpert for TB care, compared to a base case of smear microscopy and clinical diagnosis in India, South Africa and Uganda |
| Supply ITNs for malaria,^41–43^ Africa | 61–94 | Adding insecticide-treated nets in Democratic Republic of Congo, western Kenya and Tanzania |
| Rural trauma hospital ^44^ | 87 | Modelled based on costs and estimated DALYs saved for all admissions over a three-month period for a trauma hospital in Cambodia, excluding outpatients |
| Home presumptive treatment malaria,^45^ Africa | 93 | Treating fever at home with artesunate and amodiaquine |
| **Figure 2** | | |
| Preventive chemotherapy for schistosomiasis and STHs^46^ | 114 | Integrated Mass Drug Administration for schistosomiasis and soil-transmitted helminthiasis in four Communities in Cote d’Ivoire |
| Elimination of Mother-To-Child Transmission (EMTCT) Option B HIV vs Option A,^47,48^ Africa | 65–251 | Long-course ART prophylaxis for pregnant women in Nigeria and Uganda. |
| Primary prevention of ARF/RHD, children with GAS pharyngitis^49^ | 135 | Treatment with intramuscular penicillin for South African children presenting with CDR score higher than two |
| Elimination of Mother-To-Child Transmission (EMTCT) Option A HIV vs no treatment,^50–53^ Africa | 26–730 | Short-course EMTCT program versus no treatment |
| Add syphilis screen to HIV screen/treat,^54^ UMIC | 140 | Adding syphilis screen and treat to HIV screen and treat program in China |
| Beta-blocker and ACE inhibitor vs no medication, heart failure, access to treatment^3^ | 124–219 | Regimen consisted of a beta-blocker and an ACE-inhibitor was  given to patients with a history of CVD to prevent CVD, with access to treatment |
| Scale up ART to all <350, or all infected,^55,56^ S Af | 188–256 | Scaling up access to antiretroviral therapy (ART) for all identified HIV-infected individuals in South Africa |
| Treat breast cancer MIC^57^ | 230 | Treat (vs not treat) cancer at any stage in Mexico |
| *HPV (Human Papillomavirus) vaccination @$50/girl MIC^58,59^ | 198–296 | HPV vaccine of young girls in Brazil, 70% vaccine coverage. Converted from QALY’s. |
| Trauma center^60^ | 218–302 | Modelled based on costs and estimated DALYs saved associated with all admissions for a trauma hospital over a three-month period. A higher proportion in Nigeria was life-saving surgery; Haiti includes burns. |
| Treat TB with second-line drugs MIC^61^ | 264 | Systematic review of treatment for Multidrug-Resistance Tuberculosis (TB) |
| Screen/treat for syphilis UMIC^62,54^ | 200–369 | Program for preventing mother-to-child transmission of syphilis in China |
| Beta-blocker and ACE inhibitor vs no med, heart failure, no access to treatment^3^ | 274 | Regimen consisted of a beta-blocker and an ACE-inhibitor was  given to patients with a history of CVD to prevent CVD, without access to treatment |
| Older anti-epileptic drug in primary care MIC^63^ | 279 | Older anti-epileptic drug in primary care in Nigeria: 50% coverage |
| Intrapartum care^64–66^ | 211–492 | Maternal health policy model used to evaluate a package of care that includes safe abortion and surgical treatment of emergency obstetric care |
| Eradicate yaws (detect and treat)^67^ | 324 | Four yaws eradication pilot sites and other mars treatment campaigns in 12 endemic countries in 2015-2020 |
| EMTCT Option A HIV vs no treatment,^50^ SE Asia | 355 | Prophylaxis provided to mothers and to child, if delivered in a healthcare facility  Elimination of Mother-To-Child Transmission: option A is single-drug regimen provided to mother during pregnancy and lactation |
| EMTCT (Elimination of Mother-To-Child Transmission) Option B+ HIV versus Option A,^68,48^ Africa | 251–502 | Lifetime multiple-drug antiretroviral therapy for pregnant women |
| Treat colorectal cancer (CRC),^69^ LIC | 430 | Treat CRC in WHO region AFR-E, 95% coverage |
| Maintenance psychosocial care for depression, primary care,^70^ UMIC | 437 | Maintenance treatment with fluoxetine and cognitive behavioural therapy for major depression in Thailand over five years |
| Non-emergency orthopaedic conditions^71,72^ | 359–540 | Volunteer surgical missions in Dominican Republic and Nicaragua, (knee osteoarthritis, fractures, dislocations, amputations, injured nerves and congenital malformations) |
| BCC plus regulation, sex establishments,^73^ LAC | 557–570 | Interventions include community mobilization, promotional media, interpersonal communication and counseling, implemented in female sex establishments in the Dominican Republic |
| Secondary prevention (medication) CVD vs no treatment^74^ | 570–970 | Secondary prevention consists of giving aspirin, statin and  a calcium-channel blocker to patients with a history of CVD |
| Episodic psychosocial care for depression, primary care,^70^ UMIC | 914 | Episodic treatment with fluoxetine and cognitive behavioural therapy for major depression in Thailand |
| **Figure 3** | | |
| HPV (Human papillomavirus) vaccination @$240+/girl^75–77^ | 168–5168 | HPV vaccination at age 12 in Thailand, 100% compliance; uses QALYs |
| Use Xpert to diagnose TB, MIC^78^ | 810–1316 | Implementation of Xpert for Tuberculosis diagnosis in five southern African countries, compared to current diagnostic strategy of sputum smear |
| BCC (Behavior Change Communications) alone, sex establishments,^73^ LAC | 1186 | Interventions include community mobilization, promotional media, interpersonal communication and counseling, implemented in female sex establishments in the Dominican Republic |
| Primary prevention CVD abs risk >40% UMIC^3^ | 1373 | Antihypertensive therapy was given to individuals in South Africa  having an absolute risk of CVD over the next ten years of 40% |
| Facility-based treatment of schizophrenia with drugs, MIC^63,79^ | 1427–1574 | Treat schizophrenia with anti-psychotic and psychosocial treatment in psychosocial care center |
| Telemedicine diabetic retinopathy screening, 1-2 times/lifetime MIC^80^ | 1605 | Screening once or twice a lifetime versus no screening |
| Treatment of depression in primary care with drugs, MIC^63,70^ | 1312–2048 | Treatment with anti-depressant and cognitive behavioural therapy in primary healthcare |
| Screen and treat breast cancer MIC^57^ | 1838 | Mammography and treat (vs neither) in Mexico |
| Primary prevention CVD with four drugs MIC^74,81^ | 1070–3207 | Patients without a history of CVD were given aspirin and statin, vs no medication |
| Vector control for dengue^82^ | 2500–3000 | 43 insecticide-based vector control strategies including larval and adult control for a five-year period |
| Online sex education to prevent sexually transmitted infections^83^ | 1180–10256 | Online sexual-health education course targeting ninth- grade students in Colombian urban public secondary schools |
| Screen and treat breast cancer LIC^84^ | 3578 | Biennial mammography for women aged 40-69 + treat all (vs not) in Ghana |
| EMTCT Option A (with mass screening) versus no treatment,^85^ LAC | 3092–7924 | Elimination of Mother-To-Child Transmission (Counselling pre and post test, and nevirapine for mother and child if accepted) |
| PrEP-ARV for non-infected partner, serodiscordant couples^86^ | Cost-saving to 6468 | PrEP-ARV (pre-exposure prophylaxis with antiretrovials) for non-infected partner, serodiscordant couples |
| **Figure 4** | | |
| Treat severe malaria with artesunate versus quinine,^7,8^ Africa & Southeast Asia | 5 | Use of parenteral artesunate to treat children with severe malaria in Africa and Southeast Asia |
| Zinc added to oral rehydration therapy^87^ | 10–50 | Used zinc as adjunct therapy to standard treatment of acute childhood diarrhea |
| Community management severe-acute malnutrition^97–99^ | 25–40 | Community-based therapeutic care: Diagnosis, RUTF (Ready-to-Use-Therapeutic Food), supplements, in-patient treatments, out-patient visits, weekly follow ups |
| Maternal and neonatal care at home^88^ | 13–126 | Maternal and neonatal services delivered at home, with community mobilization and health system strengthening |
| Micronutrient interventions (biofortification, fortification, supplementation)^89–96^ | 20–100 | Vitamin A supplementation, fortification and biofortification, zinc supplementation and fortification, and iron, folic acid and Vitamin B12 fortification |
| Management of obstructed labour^100^ | 77 | Skilled attendance at birth, offering first level maternal and neonatal care around childbirth, emergency obstetric and neonatal care around and after birth |
| Clean delivery kit and train TBAs^88^ | 82 | Training traditional birth attendants (TBAs) to perform interventions targeting birth asphyxia, hypothermia and neonatal sepsis |
| Education programmes on nutrition/WASH^101^ | 95 | Health facility-based nutrition/water and sanitation education program targeting children under 2 years; converted from deaths-averted |
| Home management of fever with antimalarials versus referral^45^ | 96 | Treat fever with artesunate-amodiaquine |
| Original EPI-6 (Expanded Program of Immunization with six vaccines) plus Hepatitis B^103,37–39^ | 103 | Infant immunization |
| Pneumococcus and rotavirus,^104–116^ LIC | 103 | Implementing pneumococcus and rotavirus vaccination program; low income countries are eligible to procure vaccines from Gavi at low prices |
| Handwashing BCC (behavior change communications)^102^ | 90–225 | Increase hand-washing after handling child stool and disposal of stool in latrines |
| Oral rehydration therapy^87^ | 153 | Standard case management of acute childhood diarrhea with oral rehydration salts |
| Household water treatment, LIC^120^ | 190 | Household chlorination |
| Access to modern contraceptives^119^ | 150–300 | Universal access to modern contraceptives |
| Quality improvement protocol newborns in hospital^88^ | 305 | Used facility data to monitor indicators of common technical interventions. Staff worked collaboratively to identify strategies to overcome service delivery barriers and improve facility care |
| Intrapartum care in Latin America and the Caribbean^64^ | 310 | Intrapartum care and comprehensive emergency obstetric care |
| Intrapartum care,^65,66,121^ LICs* | 200–500 | Facility-based birth with Traditional Birth Attendants, transport from home to referral facility,  transport from facility to emergency obstetrical care or from basic to comprehensive emergency obstetric care, availability of quality emergency obstetric care and facility expedient quality care |
| Comprehensive nutrition package (all interventions Lancet 2013) | 353 | Interventions addressing undernutrition and micronutrient deficiencies in women and children in 34 countries |
| *Mother's groups to improve maternal/neonatal health^117,118^ | 150–1000 | Mother’s group to improve maternal and neonatal health in Bangladesh and India; converted from LYS |
| HiB and rubella added to EPI, ^105,123–128^ LICs | 368–768 | Introduction of HiB and rubella into DPT-Hepatitis B program |
| Region specific vaccines (Yellow fever, Japanese encephalitis, meningitis A)^129–131^ | 368–768 | Japanese encephalitis vaccination program for 9 month olds (routine immunization) (at US$0.30 per dose), reactive meningococcal vaccination (at US$0.64 per dose), |
| Pneumococcus and rotavirus (market price, lower-mid income countries)^104,105,111,113,132–140^ | 368–768 | Add two vaccines to national vaccination program |
| Cholera and typhoid vaccination^141,142^ | 2018 | School-based vaccination program targeting school children (5-14 years) |
| Pneumococcus and rotavirus (market price, upper-mid IC)^113,114,133,135,143–146^ | 2018 | Adding pneumococcus and rotavirus vaccination to standard vaccinations |
| C-section, all LMICs^147^ | 1600–2600 | Treating obstructed labor with Caesarean delivery in 49 countries, across multiple regions, identified by the WHO |
| Rural water supply/sanitation, LIC^148^ | 2200 | Piped water supply and sewer connection, 98% coverage |
| Urban water supply/sanitation, LIC^148^ | 2900 | Piped water supply and sewer connection, 98% coverage |
| Microfinance/gender training for intimate partner violence^149^ | 2910 | Microfinance with gender and HIV training in four villages in South Africa |

Abbreviations

CVD = cardiovascular disease

LIC = low income country/countries

MIC = middle income country/countries

LMIC = low and middle income country/countries

UMIC = upper-middle income country/countries

**References.**

1. Wang X, Li W, Li X, et al. Effects and cost-effectiveness of a guideline-oriented primary healthcare hypertension management program in Beijing, China: results from a 1-year controlled trial. Hypertens Res 2013; 36: 313–21.
2. Rubinstein A, Colantonio L, Bardach A, et al. Estimation of the Burden of Cardiovascular Disease Attributable to Modifiable Risk Factors and Cost-Effectiveness Analysis of Preventative Interventions to Reduce This Burden in Argentina. BMC Public Health 2010; 10: e627.
3. Gaziano TA, Steyn K, Cohen DJ, Weinstein MC, Opie LH. Cost-Effectiveness Analysis of Hypertension Guidelines in South Africa: Absolute Risk versus Blood Pressure Level. Circ 2005; 112: 3569–76.
4. Marseille E, Kahn JG, Billinghurst K, Saba J. Cost-Effectiveness of the Female Condom in Preventing HIV and STDs in Commercial Sex Workers in Rural South Africa*. Soc Sci Med* 2001; 52: 135–48.
5. Keating J, Yukich JO, Mollenkopf S, Tediosi F. Lymphatic filariasis and onchocerciasis prevention, treatment, and control costs across diverse settings: A systematic review. Acta Trop 2014; 135: 86–95
6. Turner HC, Walker M, Churcher TS, et al. Reaching the London Declaration on Neglected Tropical Diseases Goals for Onchocerciasis: An Economic Evaluation of Increasing the Frequency of Ivermectin Treatment in Africa. Clin Infect Dis 2014; 59: 923–32.
7. Lubell Y, Riewpaiboon A, Dondorp AM, et al. Cost-effectiveness of parenteral artesunate for treating children with severe malaria in sub-Saharan Africa. Bull World Health Organ 2011; 89: 504–512.
8. Lubell Y, Yeung S, Dondorp AM, et al. Cost-Effectiveness of Artesunate for the Treatment of Severe Malaria. Trop Med Int Health 2009; 14: 332–37.
9. Ferrante D, Konfino J, Mejia R, et al. The Cost-Utility Ratio of Reducing Salt Intake and Its Impact on the Incidence of Cardiovascular Disease in Argentina. Revista Panamericana de Salud Pública 2012; 32: 274–80.
10. Murray CJ, Lauer JA, Hutubessy RCW, et al. Effectiveness and Costs of Interventions to Lower Systolic Blood Pressure and Cholesterol: A Global and Regional Analysis on Reduction of Cardiovascular-Disease Risk. Lancet 2003; 361: 717–25.
11. Auvert, B, Marseille E, Korenromp EL, et al. Estimating the Resources Needed and Savings Articipated from Roll-Out of Adult Male Circumcision in Sub-Saharan Africa. PLoS ONE 2008; 3: e2679.
12. Fieno JV. Costing adult male circumcision in high HIV prevalence, low circumcision rate countries. AIDS Care 2008; 20: 515–520.
13. Uthman OA, Popoola TA, Uthman MMB, Aremu O. Economic evaluations of adult male circumcision for prevention of heterosexual acquisition of HIV in men in Sub-Saharan Africa: A systematic review. PLoS ONE 2010; 5**:** e9628.
14. Schackman BR, Neukermans CP, et al. Cost-Effectiveness of Rapid Syphilis Screening in Prenatal HIV Testing Programs in Haiti. PLoS Med 2007; 4: e183
15. McCord C, Chowdhury Q. A cost effective small hospital in Bangladesh: what it can mean for emergency obstetric care. Int J Gynaecol Obstet 2003; 81: 83-92.
16. Schulman-Marcus J, Prabhakaran D, Gaziano T. Pre-Hospital ECG for Acute Coronary Syndrome in Urban India: A Cost-Effectiveness Analysis. BMC Cardiovasc Disord 2010: 10: e13.
17. Kuznik A, Lamorde M, Nyabigambo A, Mananabe YC. Antenatal Syphilis Screening Using Point-of-Care Testing in Sub-Saharan African Countries: A Cost-Effectiveness Analysis. PLoS Med 2013; 10: e1001545.
18. Sutherland CS, Yukich Y, Goeree R, Tediosi F. A Literature Review of Economic Evaluations for a Neglected Tropical Disease: Human African Trypanosomiasis (“Sleeping Sickness”). PLoS Negl Trop Dis 2015*;* 9: e0003397.
19. Borgdorff MW, Floyd K,. Broekmans.JF. Interventions to reduce tuberculosis mortality and transmission in low- and middle-income countries. Bull World Health Organ 2002; 80: 217–227.
20. Lansingh VC, Carter MJ, Martens M. Global cost-effectiveness of cataract surgery. Ophthalmol 2007; 114: 1670–78.
21. Federici C, Fitzpatrick C, Be-Nazir A, Meheus F, Dagne D. The cost-effectiveness of a comprehensive programme to eliminate visceral leishmaniasis in Bangladesh. Draft, 2016.
22. Coleman PG, Morel C, Shillcutt S, Goodman C, Mills AJ. A threshold analysis of the cost-effectiveness of artemisinin-based combination therapies in sub-saharan Africa. Am J Trop Med Hyg 2004; 71: 196–204.
23. Orlando S, Marazzi MC, Mancinelli S et al. Cost-Effectiveness of Using HAART in Prevention of Mother-to-Child Transmission in the DREAM-Project Malawi. J AIDS 2010; 55: 631–14.
24. Corlew D. Estimation of impact of surgical disease through economic modeling of cleft lip and palate care. World J Surg 2010; 34: 391–96.
25. Magee WP Jr., Vander Burg R, Hatcher KW. Cleft lip and palate as a cost-effective health care treatment in the developing world. World J Surg 2010; 34: 420–427.
26. Moon W, Perry H, Baek RM. Is international volunteer surgery for cleft lip and cleft palate a cost-effective and justifiable intervention? A case study from East Asia. World J Surg 2012; 36: 2819–30.
27. Shillcutt SD, Clarke MG, Kingsnorth AN. Cost-effectiveness of groin hernia surgery in the western region of Ghana. Arch Surg 2010; 145: 954–961.
28. Shillcutt S, Sanders D, Butrón-Vila MT, Kingsnorth A. Cost-effectiveness of inguinal hernia surgery in northwestern Ecuador. World J Surg 2013; 37: 32–41.
29. Conteh L, Patouillard E, Kweku M, Legood R, Greenwood B, Chandramohan D. Cost effectiveness of seasonal intermittent preventive treatment using amodiaquine & artesunate or sulphadoxine-pyrimethamine in Ghanaian children. PloS ONE 2010; 5: e12223.
30. Ross A, Maire N, Sicuri E, Smith T, Conteh L. Determinants of the cost-effectiveness of intermittent preventive treatment for malaria in infants and children. PLoS ONE 2011; 6: e18391.
31. Baltussen R, Smith A. Cost effectiveness of strategies to combat vision and hearing loss in sub-Saharan Africa and South East Asia: mathematical modelling study. BMJ 2012; 344: e615.
32. Baltussen RMPM, Sylla M, Frick KD, Mariotti SP. Cost-effectiveness of trachoma control in seven world regions. Ophthalmic Epidemiol 2005; 12: 91–101.
33. Mbonye AK, Hansen KS, Bygbjerg IC, Magnussen P. Intermittent preventive treatment of malaria in pregnancy: the incremental cost-effectiveness of a new delivery system in Uganda. Trans R Soc Trop Med Hyg 2008; 102: 685–693.
34. Remme JHF, Feenstra P, Lever PR, et al. Tropical Diseases Targeted for Elimination: Chagas Disease, Lymphatic Filariasis, Onchocerciasis, and Leprosy. In: Jamison D.T., J.G. Breman, A. R. Measham, M. Claeson, D.B.Evans, P. Jha, A.R. Measham and A. Mills., editors. Disease Control Priorities in Developing Countries. 2nd edition. Washington (DC): World Bank; 2006: 433–449.
35. Yukich J, Tediosi F, Lengeler C, et al. Costs and consequences of large-scale vector control for malaria. Malaria J 2008: 7: 258 Doi: 10.1186/1475-2875-7-258
36. Utzinger J, Tozan Y, Singer BH. Efficacy and cost-effectiveness of environmental management for malaria control. Trop Med Int Health 2001; 6: 677–687.
37. Prakash C. Crucial factors that influence Cost-effectiveness of Universal Hepatitis B Immunization in India. Int J Health Technol Assess Health Care 2003; 19: 28–40.
38. Griffiths UK, Hutton G, Das Doares Pascoal E. The cost-effectiveness of introducing hepatitis B vaccine into infant immunization services in Mozambique. Health Policy Plan 2005: 20: 50–9.
39. Kim SY, Salomon JA, Goldie SJ. Economic evaluation of hepatitis B vaccination in low-income countries: using cost-effectiveness affordability curves. Bull World Health Organ 2007; 85: 833–42.
40. Vassall A, van Kampen S, Sohn H, et al. Rapid diagnosis of tuberculosis with the Xpert MTB/RIF assay in high burden countries: a cost-effectiveness analysis. PLoS Med 2011; 8: e1001120.
41. Becker-Dreps SI, Biddle AK, Pettifor A, et al. Cost-effectiveness of adding bed net distribution for malaria prevention to antenatal services in Kinshasa, Democratic Republic of the Congo. Am J Trop Med Hyg 2009; 81:496–502.
42. Wiseman V, Hawley WA, Terkule RO, et al. The cost-effectiveness of permethrin-treated bed nets in an area of intense malaria transmission in western Kenya. Am J Trop Med Hyg 2003; 68 (Suppl. 4): S161–167.
43. Hanson K, Kikumbih KN, Armstrong Schellenberg J, et al. Cost-effectiveness of social marketing of insecticide-treated nets for malaria control in the United Republic of Tanzania. Bull World Health Organ 2003; 81: 267–276.
44. Gosselin RA, Heitto M. Cost-effectiveness of a district trauma hospital in Battambang, Cambodia. World J Surg 2008; 32: 2450–53.
45. Nonvignon, J, Chinbuah MA, Gyapong M, et al. Is Home Management of Fevers a Cost-Effective Way of Reducing Under-Five Mortality in Africa? The Case of a Rural Ghanaian District. Trop Med Int Health 2012; 17: 951–57.
46. Lo NC, Bogoch II, Blackburn BG, et al. Comparison of community-wide, integrated mass drug administration strategies for schistosomiasis and soil-transmitted helminthiasis: a cost-effectiveness modelling study. Lancet Glob Health 2015; 3: e629–38.
47. Shah M, Johns B, Abimiku A, Walker DG. Cost-Effectiveness of New WHO Recommendations for Prevention of Mother-to-Child Transmission of HIV in a Resource-Limited Setting. AIDS 2011; 25: 1093–102.
48. Kuznik A, Lamorde M, Hermans S, et al. Evaluating the Cost-Effectiveness of Combination Antiretroviral Therapy for the Prevention of Mother-to-Child Transmission of HIV in Uganda. Bull World Health Organ 2012; 90: 595–603.
49. Irlam, J, Mayosi BM, Engel M, Gaziano TA. Primary Prevention of Acute Rheumatic Fever and Rheumatic Heart Disease with Penicillin in South African Children with Pharyngitis: A Cost-Effectiveness Analysis. Circ Cardiovasc Qual Outcomes 2013; 6: 343–51
50. Hogan DR, Baltussen R, Hayashi C, Lauer JA, Salomon JA. Cost Effectiveness Analysis of Strategies to Combat HIV/AIDS in Developing Countries. BMJ 2005; 331: 1431–37.
51. Marseille E, Kahn JG, Mmiro F, et al. The Cost Effectiveness of a Single-Dose Nevirapine Regimen to Mother and Infant to Reduce Vertical HIV-1 Transmission in Sub-Saharan Africa. Ann N Y Acad Sci 2000; 918: 53–56.
52. Wilkinson D, Floyd K, Gilks CF. National and Provincial Estimated Costs and Cost Effectiveness of a Programme to Reduce Mother-to-Child HIV Transmission in South Africa. South African Med J 2000; 90: 794–98.
53. Sweat MD, O'Reilly KR, Schmid GP, Denison J,de Zoysa I. Cost-Effectiveness of Nevirapine to Prevent Mother-to-Child HIV Transmission in Eight African Countries. AIDS 2004; 18: 1661–71.
54. Owusu-Edusei K Jr., Tao G, Gift TL, et al. Cost-Effectiveness of Integrated Routine Offering of Prenatal HIV and Syphilis Screening in China. Sex Transm Dis 2014; 41: 103–10.
55. Alistar SS, Grant PM, Bendavid E. Comparative Effectiveness and Cost-Effectiveness of Antiretroviral Therapy and Pre-Exposure Prophylaxis for HIV Prevention in South Africa. BMC Med 2014; 12: e11.
56. Granich R, Kahn JG, Bennett R, et al. Expanding ART for Treatment and Prevention of HIV in South Africa: Estimated Cost and Cost-Effectiveness 2011–2050. PLoS ONE 2012; 7**:** e30216.
57. Salomon, JA, Carvalho N, Gutierrez-Delgado C, et al. Intervention Strategies to Reduce the Burden of Non-Communicable Diseases in Mexico: Cost-Effectiveness Analysis. BMJ 2012; 344: e355.
58. Kawai K, de Araujo GTB, Fonseca, Pillsbury M, Singhal PK. Estimated health and economic impact of quadrivalent HPV (types 6/11/16/18) vaccination in Brazil using a transmission dynamic model. BMC Infect Dis 2012; 12: e250.
59. Vanni T, Mendes LP, Foss A, Mesa-Frias M, Legood R. Economic Modelling Assessment of the HPV Quadrivalent Vaccine in Brazil: A Dynamic Individual-based Approach. Vaccine 2012; 30: 4866–71.
60. Gosselin RA, Maldonado A, Elder G. Comparative cost-effectiveness analysis of two MSF surgical trauma centers. World J Surg 2010; 34: 415–19.
61. Fitzpatrick MC, Floyd K. A systematic review of the cost and cost effectiveness of treatment for multidrug-resistant tuberculosis. Pharmacoeconomics 2012; 30: 63–80.
62. Hong FC, Liu JB, Feng TJ. Congenital Syphilis: An Economic Evaluation of a Prevention Program in China. Sex Transm Dis 2010; 37: 26–31.
63. Gureje O, Chisholm D, Kola L, Lasebikan V, Saxena S. Cost-effectiveness of an essential mental health intervention package in Nigeria. World Psychiatry 2007: 6: 42–8.
64. Hu D, Bertozzi SM, Gakidou E, Sweet S, Goldie S. The costs, benefits, and cost-effectiveness of interventions to reduce maternal morbidity and mortality in Mexico. PLOS ONE 2007; doi 10.1371/journal.pone.0000750.
65. Goldie SJ, Sweet S, Carvalho N, Natchu UC, Hu D. Alternative strategies to reduce maternal mortality in India: a cost-effectiveness analysis. PLoS Med 2010; 7: e1000264.
66. Carvalho N, Salehi AS, Goldie SJ. National and sub-national analysis of the health benefits and cost-effectiveness of strategies to reduce maternal mortality in Afghanistan. Health Policy Plan 2013; 28: 62–74.
67. Fitzpatrick C, Asiedu K, Jannin J. Where the Road Ends, Yaws Begins? The Cost-effectiveness of Eradication versus More Roads. PLoS Negl Trop Dis 2014; 8: e3165.
68. Robberstad B, Evjen-Olsen B. Preventing Mother to Child Transmission of HIV with Highly Active Antiretroviral Treatment in Tanzania—A Prospective Cost-Effectiveness Study. J AIDS 2010; 55: 397–403.
69. Ginsberg GM, Lauer JA, Zelle S, Baeten S, Baltussen R. Cost effectiveness of strategies to combat breast, cervical, and colorectal cancer in sub-Saharan Africa and South East Asia: mathematical modeling study. BMJ 2012: 344: e614.
70. Prukkanone B, Vos T, Bertram M, Lim, S. Cost-effectiveness analysis for antidepressants and cognitive behavioral therapy for major depression in Thailand. Value Health 2012; 15: S3-S8.
71. Gosselin RA, Gialamas G, Atkin DM. Comparing the cost-effectiveness of short orthopedic missions in elective and relief situations in developing countries. World J Surg 2011; 35: 951–55.
72. Chen AR, Pedtke A, Kobs JK, et al. Volunteer orthopedic surgical trips in Nicaragua: a cost-effectiveness evaluation.” World J Surg 2012; 35: 951-955.
73. Sweat M, Kerrigan K, Moreno L, el al Cost-Effectiveness of Environmental-Structural Communication Interventions for HIV Prevention in the Female Sex Industry in the Dominican Republic. J Health Communication 2006; 11 (Suppl 2): S123–42
74. Gaziano TA, Opie LH, Weinstein MC. Cardiovascular Disease Prevention with a Multidrug Regimen in the Developing World: A Cost-Effectiveness Analysis. Lancet 2006; 368 : 679–86.
75. Insinga RP, Dasbach EJ, Elbasha EH, Puig A, Reynales-Shigematsu LM. Cost-effectiveness of Quadrivalent Human Papillomavirus (HPV) Vaccination in Mexico: A Transmission Dynamic Model-based Evaluation. Vaccine 2007; 26: 128–39.
76. Praditsitthikorn N, Teerawattananon Y, Tantivess S, et al. Economic Evaluation of Policy Options for Prevention and Control of Cervical Cancer in Thailand. Pharmacoeconomics 2011; 29**:** 781–806.
77. Termrungruanglert W, Havanond P, Khemapech N, et al. Model for Predicting the Burden and Cost of Treatment in Cervical Cancer and HPV-related Diseases in Thailand. Eur J Gynaecol Oncol 2012*:* 33: 391–94.
78. Menzies NA, Cohen T, Lin HH, Murray M, Salomon JA. Population health impact and cost-effectiveness of tuberculosis diagnosis with Xpert MTB/RIF: a dynamic simulation and economic evaluation. PLoS Med 2012; 9: e1001347.
79. Lindner LM, Marasciulo AC, Farias MR, Grohs GEM. Economic evaluation of antipsychotic drugs for schizophrenia treatment within the Brazilian Healthcare System. Revista de saúde pública 2009; 43: 62 .
80. Rachapelle S, Legood R, Alavi Y, et al. The Cost-Utility of Telemedicine to Screen for Diabetic Retinopathy in India. Ophthalmology 2013; 120: 566–73.
81. Rubinstein A, Garcia Marti S, Souto A, Ferrante D, Augustovski F. Generalized Cost-Effectiveness Analysis of a Package of Interventions to Reduce Cardiovascular Disease in Buenos Aires, Argentina. Cost Eff Resource Alloc 2009; 7: 10.
82. Luz PM, Vanni T, Medlock J, Paltiel AD, Galvani AP. Dengue vector control strategies in an urban setting: an economic modelling assessment. *Lancet* 2011; **377**: 1673–80.
83. Chong A, Gonzalez-Navarro M, Karlan D, Valdivia M. Effectiveness and Spillovers of Online Sex Education: Evidence from a Randomized Evaluation in Colombian Public Schools. Working Paper 18776, National Bureau of Economic Research*,* Cambridge, MA, 2013*.* Available: <http://www.nber.org/papers/w18776>. Accessed: July 3 2016.
84. Zelle SG, Nyarko KM, Bosu WK, et al. Costs, Effects and Cost-Effectiveness of Breast Cancer Control in Ghana Tropical Medicine and International Health. Trop Med Int Health 2012; 17: 1031–1043.
85. Aldridge RW, Iglesias D, Caceres CF, Miranda JJ. Determining a Cost Effective Intervention Response to HIV/AIDS in Peru. BMC Public Health 2009; 9**:** e352.
86. Hallett TB, Baeten JM, Heffron R, et al. Optimal uses of antiretrovirals for prevention in HIV-1 serodiscordant heterosexual couples in South Africa: a modelling study. PLoS Med 2011; 8: e1001123. doi:10.1371/journal.pmed.1001123
87. Robberstad, B, Strand T, Black RE, Sommerfelt H. Cost-effectiveness of zinc as adjunct therapy for acute childhood diarrhoea in developing countries. Bull World Health Organ 2004; 82: 523–31.
88. Mangham-Jefferies L, Pitt C, Cousens S, Mills A, Schellenberg J. Cost-effectiveness of strategies to improve the utilization and provision of maternal and newborn health care in low-income and lower-middle-income countries: a systematic review. BMC Pregnancy Childbirth 2014; 14: e243.
89. Chow J., Klein EY, Laxminarayan R. Cost-effectiveness of "golden mustard" for treating vitamin A deficiency in India. PloS ONE 2010; 5: e12046.
90. Edejer TT, Aikins M, Black R, Wolfson L, Hutubessy R, Evans DB. Cost effectiveness analysis of strategies for child health in developing countries. BMJ 2005; 331: e1177.
91. Fiedler JL, Macdonald B. A strategic approach to the unfinished fortification agenda: feasibility, costs, and cost-effectiveness analysis of fortification programs in 48 countries. Food Nutr Bull 2009; 30: 283–316.
92. Ma G, Jin Y, Li Y, et al. Iron and zinc deficiencies in China: what is a feasible and cost-effective strategy? Public Health Nutr 2008; 11: 632–8.
93. Meenakshi J, Johnson N, Manyong VM, et al. How Cost-Effective is Biofortification in Combating Micronutrient Malnutrition? An Ex ante Assessment. World Development 2010; 38: 64–75.
94. Sharieff W, Horton SE, Zlotkin S. Economic gains of a home fortification program: evaluation of "Sprinkles" from the provider's perspective. Can J Public Health 2006; 97: 20–3.
95. Stein AJ, Sachdev H, Qaim M. Potential impacts and cost-effectiveness of Golden Rice. Nature Biotechnology 2006; 24: 1200–1.
96. Stein AJ, Meenakshi JV, Qaim M, Nestel P, Sachdev HP, Bhutta ZA. Potential impacts of iron biofortification in India. Soc Sci Med 2008; 66: 1797–808.
97. Bachmann MO. Cost effectiveness of community-based therapeutic care for children with severe acute malnutrition in Zambia: decision tree model. Cost Eff Resource Alloc 2009: 7: 2.
98. Puett C, Sadler K, Alderman H, Coates J, Fiedler JL, Myatt M. Cost-effectiveness of the community-based management of severe acute malnutrition by community health workers in southern Bangladesh. Health Policy Plan 2013; 28: 386–99.
99. Wilford R, Golden K, Walker DG. Cost-effectiveness of community-based management of acute malnutrition in Malawi. Health Policy Plan 2012; 27: 127–37.
100. Adam,T, Lim SS, Mehta S, et al. Cost effectiveness analysis of strategies for maternal and neonatal health in developing countries. BMJ 2005; 331: 1107.
101. Waters HR, Penny ME, Creed-Kanashiro HM, et al. The cost-effectiveness of a child nutrition education programme in Peru. Health Policy Plan 2006; 21: 257–64.
102. Borghi J, Guinness L, Ouedraogo J, Curtis V. Is hygiene promotion cost-effective? A case study in Burkina Faso. Trop Med Int Health 2002; 7: 960–9.
103. Brenzel L, Wolfson LJ, Fox-Rushby J, Miller M, Halsey NA. Vaccine-preventable diseases. In Jamison, DT Alleyne G, Breman J et al (eds.). Disease Control Priorities in Developing Countries (2nd ed.), New York: Oxford University Press, 2006: 389-411.
104. Kim SY, Lee G, Goldie SJ. Economic evaluation of pneumococcal conjugate vaccination in The Gambia. BMC Infect Dis 2010; 10: e260.
105. Niessen NW, ten Hove A, Hilderink H, Weber M, Mulholland K, Ezzati M. Comparative impact assessment of child pneumonia interventions. Bull World Health Organ 2009; 87: 472–80.
106. Tate JE, Kisakye A, Mugyenyi P, Kizza D, Odiit A, Braka F. Projected health benefits and costs of pneumococcal and rotavirus vaccination in Uganda. Vaccine 2011; 29: 3329–34.
107. Touray MM, Hutubessy R, Acharya A. The cost effectiveness of pneumococcal conjugate vaccine in the routine infant immunisation programme of The Gambia. J Pharm Health Serv Res 2011; 2: 175–84.
108. Abbott C, Tiede B, Armah G, Mahmoud A. Evaluation of cost-effectiveness of live oral pentavalent reassortant rotavirus vaccine introduction in Ghana. Vaccine 2012; 30: 2582–7.
109. Atherly D, Dreibelbis R, Parashar UD, Levin C, Wecker J, Rheingans RD. Rotavirus vaccination: cost-effectiveness and impact on child mortality in developing countries. J Infect Dis 2009; 200 (Suppl. 1): S28–38.
110. Berry S. Johns AB, Shih C, Berry AA, Walker DG. The cost-effectiveness of rotavirus vaccination in Malawi. J Infect Dis 2010; 202 (Suppl. l): S108–15.
111. Flem ET, Latipov R, Nurmatov ZS, Xue Y, Kasymbekova KT, Rheingans RD. Costs of diarrheal disease and the cost-effectiveness of a rotavirus vaccination program in Kyrgyzstan. J Infect Dis 2009; 200 (Suppl. 1): S195–202.
112. Kim, SY, Goldie SJ, Salomon JA. Cost-effectiveness of Rotavirus vaccination in Vietnam. BMC Public Health 2009; 9: e29.
113. Podewils LJ, Antil L, Hummelman E, Bresee J, Parashar UD, Rheingans R. Projected cost-effectiveness of rotavirus vaccination for children in Asia. J Infect Dis 2005; 192 (Suppl. 1): S133–45.
114. Rheingans RD, Antil L, Dreibelbis R, Podewils LJ, Bresee JS, Parashar UD. Economic costs of rotavirus gastroenteritis and cost-effectiveness of vaccination in developing countries. J Infect Dis 2009; 200 (Suppl. 1):S16–27.
115. Smith ER, Rowlinson EE, Iniguez V, et al. Cost-effectiveness of rotavirus vaccination in Bolivia from the state perspective. Vaccine 2011; 29: 6704–11.
116. Tate JE, Rheingans RD, O'Reilly CE, el al. Rotavirus disease burden and impact and cost-effectiveness of a rotavirus vaccination program in Kenya. J Infect Dis 2009*;* 200 (Suppl. 1): S76–84.
117. Fottrell E, Azad K, Kuddus A, et al. The effect of increased coverage of participatory women's groups on neonatal mortality in Bangladesh: A cluster randomized trial. JAMA Pediatrics 2013; 167: 816–25.
118. Tripathy P, Nair N, Barnett S, et al. Effect of a participatory intervention with women's groups on birth outcomes and maternal depression in Jharkhand and Orissa, India: a cluster-randomised controlled trial. Lancet 2010; 375: 1182–92.
119. Babigumira, JB, Stergachis A, Veenstra DL, et al. Potential cost-effectiveness of universal access to modern contraceptives in Uganda. PloS ONE 2012; 7: e30735.
120. Clasen T, Haller L, Walker D, Bartram J, Cairncross S. Cost-effectiveness of water quality interventions for preventing diarrhoeal disease in developing countries. J Water Healt*h* 2007; 5: 599–608.
121. Erim DO, Resch SC, Goldie SG. Assessing health and economic outcomes of interventions to reduce pregnancy-related mortality in Nigeria. BMC Public Health 2012; 12: e786.
122. Bhutta ZA, Das JK, Rizvi A, et al. Evidence-based interventions for improvement of maternal and child nutrition: what can be done and at what cost? Lancet 2013; 382: 452–77.
123. Akumu, AO, English M, Scott JA, Griffiths UK. Economic evaluation of delivering Haemophilus influenzae type b vaccine in routine immunization services in Kenya. Bull World Health Organ 2007; 85: 511–8.
124. Broughton EI. Economic evaluation of Haemophilus influenzae type B vaccination in Indonesia: a cost-effectiveness analysis. J Public Health (Oxford Journals) 2007; 29: 441–8.
125. Gessner BD, Sedyaningsih ER, Griffiths UK, et al. Vaccine-preventable haemophilus influenza type B disease burden and cost-effectiveness of infant vaccination in Indonesia. Pediatr Infect Dis *J* 2008; 27: 438–43.
126. Babigumira JB, Morgan I, Levin A. Health economics of rubella: a systematic review to assess the value of rubella vaccination. BMC Public Health 2013; 13: e406.
127. Clark AD, Griffiths UK, Abbas SS. Impact and cost-effectiveness of Haemophilus influenzae type b conjugate vaccination in India. J Pediatr 2013; 163: S60–72.
128. Platonov AE, Griffiths UK, Voeykova MV, et al. Economic evaluation of Haemophilus influenzae type b vaccination in Moscow, Russian Federation. Vaccine 2006; 24: 2367–76.
129. Touch S, Suraratdecha C, Samnang C, et al. A cost-effectiveness analysis of Japanese encephalitis vaccine in Cambodia. Vaccine 2010; 28: 4593–9.
130. Miller MA, Shahab CK. Review of the cost effectiveness of immunisation strategies for the control of epidemic meningococcal meningitis. Pharmacoeconomics 2005; 23: 333–43.
131. Monath TP, Nasidi A. Should yellow fever vaccine be included in the Expanded Program of Immunization in Africa? A cost-effectiveness analysis for Nigeria. Am J Trop Med Hyg 1993; 48: 274–99.
132. Constenla DO, Linhares AC, Rheingans RD, Antil LR, Waldman EA, da Silva LJ. Economic impact of a rotavirus vaccine in Brazil. J Health Pop Nutr 2008; 26: 388–96.
133. Nakamura MM, Tasslimi A, Lieu TA, et al. Cost effectiveness of child pneumococcal conjugate vaccination in middle-income countries. Int Health 2012; 3: 270–81.
134. Sinha A, Levine O, Knoll MD, Muhib F, Lieu TA. Cost-effectiveness of pneumococcal conjugate vaccination in the prevention of child mortality: an international economic analysis. Lancet 2007; 369: 89–96.
135. Sinha A, Constenla D, Valencia JE, et al. Cost-effectiveness of pneumococcal conjugate vaccination in Latin America and the Caribbean: a regional analysis. Revista Panamericana de Salud Publica 2008; 24: 304–13.
136. Chotivitayatarakorn P, Poovorawan Y. Cost-effectiveness of rotavirus vaccination as part of the national immunization program for Thai children. Southeast Asian J Trop Med Public Health 2010; 41: 114–25.
137. Clark AD, Walker DG, Mosqueira NR, et al. Cost-effectiveness of rotavirus vaccination in Peru. J Infect Dis 2009; 200 (Suppl. 1): S114–24.
138. Constenla D, Velazquez FR, Rheingans RD, Antil L, Cervantes Y. Economic impact of a rotavirus vaccination program in Mexico. Revista Panamericana de Salud Publica 2009; 25: 481–90.
139. Jit M, Yuzbashyan R, Sahakyan G, Avagyan, Mosina L. The cost-effectiveness of rotavirus vaccination in Armenia. Vaccine 2011; 29: 9104–9111.
140. Rheingans RD, Constenla D, Antil L, Innis BL, Breuer T. Potential cost-effectiveness of vaccination for rotavirus gastroenteritis in eight Latin American and Caribbean countries. Revista Panamericana de Salud Publica 2007: 21: 205–16.
141. Jeuland M, Cook J, Poulos C, Clemens J, Whittington D. Cost-effectiveness of new-generation oral cholera vaccines: a multisite analysis. Value Health 2009; 12: 899–908.
142. Cook J, Jeuland M, Whittington D, et al. The cost-effectiveness of typhoid Vi vaccination programs: calculations for four urban sites in four Asian countries. Vaccine 2008; 26: 6305–16.
143. Sartori AM, de Soarez PC, Novaes HM. Cost-effectiveness of introducing the 10-valent pneumococcal conjugate vaccine into the universal immunisation of infants in Brazil. J Epidemiol Community Health 2012; 66: 210–7.
144. Uruena A, Pippo T, Sol Betelu M, et al. Cost-effectiveness analysis of the 10-and 13-valent pneumococcal conjugate vaccines in Argentina. Vaccine 2011; 29: 4963–72.
145. Vespa G, Constenla DO, Pepe C, et al. Estimating the cost-effectiveness of pneumococcal conjugate vaccination in Brazil. Revista Panamericana de Salud Publica 2009; 26: 518–28.
146. De la Hoz F, Alvis N, Narvaez J, Cediel N, Gamboa O, Velandia M. Potential epidemiological and economical impact of two rotavirus vaccines in Colombia. Vaccine 2010; 28: 3856–64.
147. Alkire BC, Vincent JR, Burns CT, Metzler IS, Farmer PE, Meara JG. Obstructed labor and caesarean delivery: the cost and benefit of surgical intervention*.* PloS O*NE* 2012; 7: e34595.
148. Haller L, Hutton G, Bartram J. Estimating the costs and health benefits of water and sanitation improvements at global level. J Water Health 2007; 5: 467–80.
149. Jan S, Ferrari G, Watts CH, et al. Economic evaluation of a combined microfinance and gender training intervention for the prevention of intimate partner violence in rural South Africa. Health Policy Plan 2011; 26: 366–72.
